# Sleep quality and psychological distress among Bangladeshi medical students: Prevalence, predictors, and sex-institutional differences

**DOI:** 10.1016/j.gloepi.2026.100243

**Published:** 2026-01-06

**Authors:** Abdul Muyeed, Ratul Rahman, Sumaiya Islam Suchi, Kawsar Ahmed, Tahmina Akter Tithi

**Affiliations:** a Department of Statistics, Jatiya Kabi Kazi Nazrul Islam University, Trishal, Mymensingh 2224, Bangladesh; b Department of Marketing, Jatiya Kabi Kazi Nazrul Islam University, Trishal, Mymensingh 2224, Bangladesh; c Ministry of Health and Family Welfare, Dhaka, Bangladesh

**Keywords:** Psychological distress, Mental health, Sleep quality, Medical students, Bangladesh

## Abstract

**Background:**

Poor **s**leep quality and psychological distress are common in medical students worldwide. Understanding the relationship between sleep quality and psychological distress is crucial for enhancing student well-being and academic achievement. This study aimed to assess the prevalence and influencing factors of poor sleep quality and psychological distress among Bangladeshi medical students, and to explore sex and institutional differences.

**Methods:**

A cross-sectional study was conducted among 378 medical students using a structured questionnaire. Data were collected using the Depression, Anxiety, and Stress Scale (DASS-21) and the Pittsburgh Sleep Quality Index (PSQI). Statistical analyses including confirmatory factor analysis (CFA), independent samples *t*-tests, and a bivariate test of association were conducted.

**Results:**

The prevalence rates of poor sleep quality (67.2 %), depression (55.8 %), anxiety (58.7 %), and stress (38.6 %) were significantly high among medical students in Bangladesh. The CFA test recommended a three-factor model for DASS-21 and a two-factor model for PSQI. A moderately positive association was found between sleep quality and depression, anxiety, and stress. Independent samples *t*-tests showed that male students reported lower PSQI and DASS-21 scores. Additionally, depression (AOR = 2.61, 95 % CI: 1.37–4.99) and stress (AOR = 2.77, 95 % CI: 1.25–6.14) were found as the most significant predictors of sleep quality.

**Conclusions:**

Psychological distress, excessive time spent on social media, and online games negatively influence sleep quality, while being a male, smoking, and having career-building opportunities positively influence sleep quality. Interventions aimed at reducing stress and promoting healthy sleep practices are urgently needed within medical institutions.

## Highlights

• High prevalence of poor sleep (67.2 %) was found among Bangladeshi medical students.
• Depression and stress were the strongest predictors of poor sleep.
• Female students reported worse sleep and higher psychological distress.
• Excessive screen time was linked to six times higher odds of poor sleep.
• Private medical students showed higher depression and anxiety.

## Introduction

Medical education is widely acknowledged as a high-pressure environment that places significant cognitive, emotional, and social demands on students. This intense training often leads to increased levels of psychological distress among medical students, including depression, anxiety, and stress, which can negatively impact their academic performance, interpersonal relationships, and overall well-being [[1], [2], [3]]. Research indicates that medical students experience higher levels of psychological distress compared to their age-matched peers in the general population, underscoring the unique vulnerability of this group [[4], [5], [6], [7]]. Contributing factors to this vulnerability include a heavy academic workload, frequent examinations, long hours of study, exposure to patient suffering, and competitive learning environments [2,3,5].

Among the various aspects of mental health, sleep quality has emerged as a critical factor influencing psychological well-being [8,9]. Insufficient or poor-quality sleep is linked to increased symptoms of depression, anxiety, stress, and even suicidal ideation [[10], [11], [12]]. Sleep disturbances are highly prevalent among medical students worldwide, with studies from South Asia reporting rates as high as 40–70 % [[13], [14], [15], [16]]. In Bangladesh, university students, including those in medical programs, often experience inadequate sleep due to academic pressures, irregular schedules, and lifestyle factors [13,[16], [17], [18]]. Poor sleep not only worsens psychological distress but also impairs cognitive functioning, memory consolidation, and clinical performance, creating a cyclical pattern that further compromises mental health [12,19].

Sex and institutional differences significantly shape the mental health profiles of medical students. Female students often report higher levels of stress and anxiety than their male counterparts. Additionally, institutional characteristics such as faculty support, access to mental health resources, and campus environment also influence psychological outcomes [1,4,20]. Research has shown that medical students in private institutions may experience different stress patterns compared to those in public institutions, likely due to variations in curriculum structure, student-faculty interaction, and the availability of wellness programs [[21], [22], [23]]. These disparities highlight the importance of considering both individual and contextual factors when assessing mental health outcomes in this population.

The theoretical model explaining psychological distress and sleep quality among medical students can be understood through the stress-diathesis model, which suggests that psychological outcomes arise from the interaction of predisposing vulnerabilities and environmental stressors [1,24]. In the context of medical education, individual factors such as personality traits, coping strategies, and previous mental health history interact with situational stressors, including academic workload, institutional environment, and social support, to influence levels of distress and sleep disruption [4,6,24]. Additionally, the biopsychosocial model offers another perspective, emphasizing how physiological factors (such as sleep and neuroendocrine responses), psychological aspects (including stress and anxiety), and social elements (like peer and faculty support) collectively affect the mental health of medical students [10,12,15]. By integrating these models, we can gain a comprehensive understanding of why some students experience significant psychological distress or sleep issues while others remain resilient.

The prevalence of psychological distress among medical students has been extensively documented worldwide. Systematic reviews and meta-analyses reveal that approximately 25–30 % of medical students' experience depression, 30–35 % suffer from anxiety, and 40–50 % report high levels of stress [2,25,26]. In Bangladesh, recent studies indicate similar trends, with a significant number of medical students showing symptoms of depression, anxiety, and insomnia [19,21,27]. Notably, the connection between sleep disturbances and psychological distress is particularly strong: students with poor sleep quality are significantly more likely to report symptoms of depression, anxiety, and stress, indicating a bidirectional relationship [12,14,16].

Several individual-level and environmental factors have been identified as predictors of psychological distress and sleep problems. Age, sex, socioeconomic status, lifestyle behaviors (such as physical activity, screen time, and caffeine consumption), and coping strategies are consistently linked to mental health outcomes [3,14,28,29]. Additionally, institutional variables including faculty support, peer networks, and the availability of mental health services affect stress levels and sleep quality [4,21,24]. These findings suggest that effective interventions should address both personal behaviors and structural conditions to alleviate distress and promote healthy sleep patterns.

**Fig. 1:**
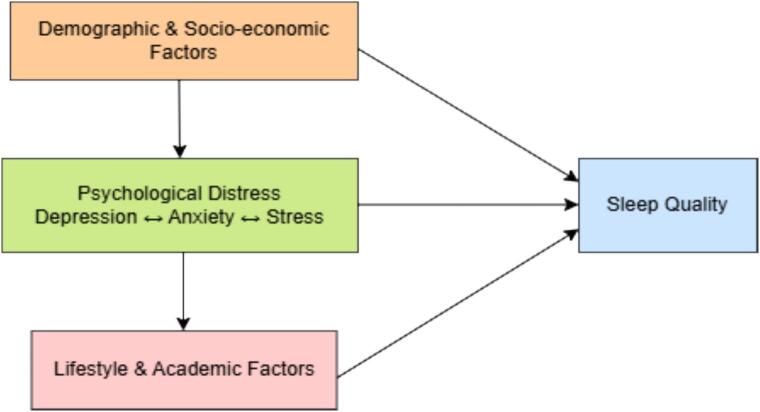
Conceptual framework illustrating the interrelationships among the factors influencing the sleep quality of medical students. Examples of demographic factors are age, height, weight, sex, religion, etc. Examples of socio-economic factors are literacy of parents, occupation of parents, etc. Examples of academic factors include daily average study hours, the result of the last professional, etc. Examples of lifestyle factors include type of medical college, average time spent on social media, etc.

In Bangladesh, the rapid expansion of medical education, combined with intense competition for admission and demanding academic expectations, heightens vulnerability to psychological distress. Students often struggle to balance their academic responsibilities with personal life, experience insufficient time for restorative sleep, and face limited access to mental health resources [27,30,31]. Additionally, the COVID-19 pandemic has introduced further challenges, such as disruptions to classroom and clinical training, social isolation, and an increased reliance on digital learning platforms, all of which have exacerbated stress and sleep-related issues [20,27]. Addressing these challenges necessitates systematic research to identify prevalence patterns, key predictors, and potential intervention targets. The study employs a conceptual framework presented in Fig. 1 that illustrates how demographic, socioeconomic, academic, and lifestyle factors collectively impact sleep quality. The framework acknowledges that antecedent factors may be interrelated and jointly influence sleep quality, thereby motivating adjustment for potential confounding variables in the regression analyses.

This study aims to investigate the prevalence of psychological distress and sleep quality issues among Bangladeshi medical students, with a particular focus on sex and institutional differences. By examining these factors within a large, representative sample, the study seeks to provide evidence for targeted interventions and institutional policies that support student well-being. Understanding the relationship between psychological distress and sleep quality through a robust theoretical framework allows for a comprehensive approach to mental health promotion in medical education. This approach not only addresses immediate well-being concerns but also contributes to long-term professional competence, as mental health during medical training is a strong predictor of future clinical performance and resilience [26,32,33].

## Materials and methods

### Study design and sampling

A cross-sectional study was conducted among the current medical students from six medical colleges in Bangladesh. The selected institutions included three government colleges; Dhaka Medical College, Mymensingh Medical College, and Khulna Medical College and three private colleges; Holy Family Red Crescent Medical College, Jahurul Islam Medical College, and TMSS Medical College. These colleges were chosen using simple random sampling from the national list of medical colleges. Within each institution, students were recruited through voluntary participation following the electronic distribution of the survey.

The required sample size was calculated using Cochran's formula, with a 95 % confidence level, a 5 % margin of error, and a prevalence estimate of 40.6 % for poor sleep quality based on previous research among medical students [34]. This value was considered in evaluating the sample size.

n=z2pqe2=1.962×0.406×0.5940.052=370.58≈371

Where, n = Sample size for medical students, z = z value (e.g., for a 95 % confidence interval, the value is 1.96), p = prevalence (40.6 %) of poor sleep quality among medical students [28], and e = Margin of error (5 %).

**Fig. 2:**
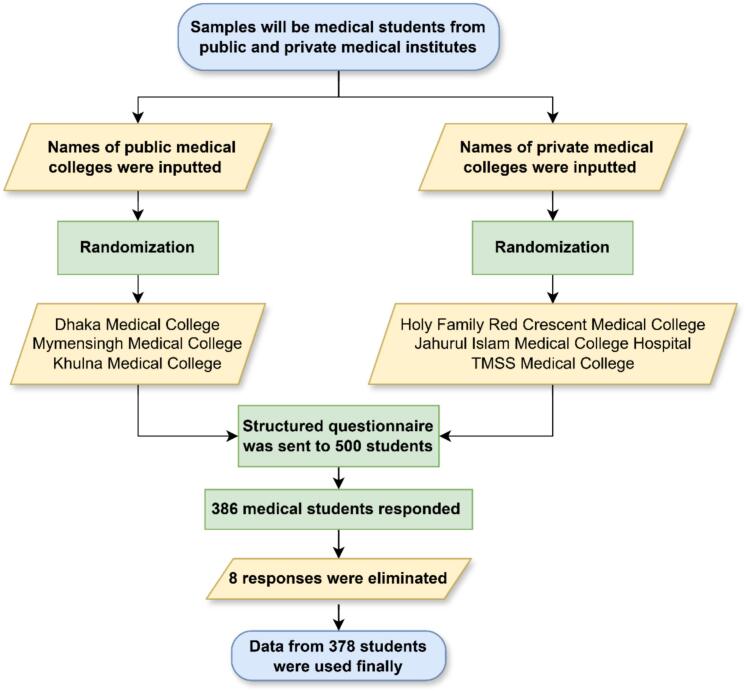
Flowchart of medical student's sampling process

Out of 500 distributed questionnaires, 386 were returned, and after excluding incomplete responses, 378 were included in the analysis. Fig. 2 represented flowchart of medical student's sampling process.

### Data collection procedures

Data collection took place between January and February 2024 using a structured self-administered questionnaire (see Supplementary Material S1) in English, hosted on Google Forms. The link was shared via institutional mailing lists, academic groups, and social media platforms. The questionnaire captured demographic and socioeconomic information, academic characteristics, and lifestyle behaviors, and included validated measures of sleep quality and psychological distress. Participation was voluntary, and informed consent was obtained electronically.

### Demographic, socio-economic, academic performance, and lifestyle-related information

This section collected data on demographic, socioeconomic, academic performance, and lifestyle-related information of the students. It contains demographic questions about age, height, weight, sex, religion, permanent residence, current residence, family type, and number of siblings. The socio-economic condition-related questions were father's education, father's occupation, mother's education, and mother's occupation, along with questions about satisfaction level with the initiatives taken by the government for medical students, opportunities for career building in Bangladesh, social value, and the professional environment. Academic performance-related questions were regarding the current professional, daily average study hours, and result of the last professional. Lastly, the survey asked about lifestyle-related questions like the type of medical college, friendly family environment, relationship status, average time spent on social media and online games, smoking status, alcohol or other drug-taking status (last three months), and whether night duty caused sleep disturbances (according to the respondent).

### Pittsburgh Sleep Quality Index (PSQI)

The Pittsburgh Sleep Quality Index (PSQI), with its 19 questions, can measure the sleep quality of the last month. The PSQI is also a widely recognized and validated instrument for measuring disturbances. The scale has also been validated among the Bangladeshi population and medical students previously [17,35]. It is a self-report questionnaire that is able to explore various dimensions of sleep, both subjective experiences and objective parameters. The PSQI has 7 components: subjective sleep quality (overall assessment of the sleep quality), sleep latency (required time to fall asleep), sleep duration (actual hours slept per night), habitual sleep efficiency (total time spent asleep and time spent in bed ratio), sleep disturbances (various sleep-related problems frequencies), use of sleeping medication, and daytime dysfunction, which have a score range of 0–3, and the summed score ranges from 0 to 21. The sum of scores for these seven components yields one global score. A score greater than 5 suggests that the respondent had significant sleep difficulties [36]. It means those who have a PSQI score of more than 5 are poor sleepers, and others are good sleepers. The higher value denotes that the problem is intense. Reliability and internal consistency of the PSQI scale were assessed by Cronbach's alpha and found to be 0.847, which shows a good internal consistency of the scale. A two-factor model containing sleep quality (which has four items: subjective sleep quality, sleep latency, sleep duration, and habitual sleep efficiency) and disturbances (which have three items: sleep disturbances, use of sleeping medication, and daytime dysfunction) was taken for confirmatory factor analysis, which was selected and tested in a previous study [37].

### Depression, Anxiety, and Stress Scale 21 (DASS-21)

The DASS-21 is a scale to evaluate emotional distress. It is the short form of the 42-item scale and is considered a self-reported scale. This scale's Bangla version has been validated among Bangladeshi university and medical students [38,39]. It has subscales for depression, anxiety, and stress separately. Every item has four rating levels, which are 0 = not at all, 1 = some of the time, 2 = good part of the time, and 3 = most of the time. Items 3, 5, 10, 13, 16, 17, and 21 are used for depression; items 2, 4, 7, 9, 15, 19, and 20 are used for anxiety; and lastly, items 1, 6, 8, 11, 12, 14, and 18 are used for stress, where the sum of the total of these seven subscales is multiplied by two to get the final scores.

Depression, anxiety, and stress have different cut-off scores. Once the scores are added up, the degree of stress (S), anxiety (A), and depression (D) is computed and explained as follows. If the combined scores for depression, anxiety, and stress are 0–9, 0–7, and 0–14, respectively, this indicates a “normal” state for each. Also, a “mild” level of D, A, and S is indicated by a 10–13, 8–9, and 15–18 score, respectively. Furthermore, “moderate” D, A, and S are indicated by scores of 14–20, 10–14, and 19–25, respectively. Additionally, D, A, and S's 21–27, 15–19, and 26–33 scores, respectively, suggest a “severe” condition. Lastly, the “extremely severe” condition is described by 28+, 20+, and 34+ of D, A, and S, respectively [40].

This recommended cut-off score represents five different states (normal, mild, moderate, severe, and extremely severe) of mental distress. Here, normal and mild stand for having no depression, anxiety, or stress separately. On the other hand, moderate, severe, and extremely severe levels represent the presence of these distresses. Other than that, extremely severe represents the highest intensity of depression, anxiety, or stress. The Cronbach alpha value of the DASS-21 scales is 0.94 in this study, which represents excellent internal consistency.

### Statistical analysis

Descriptive statistics were employed for sociodemographic variables, screen time, sleep hours, and psychological measures. Construct validity was assessed through confirmatory factor analysis (CFA), employing fit indices such as root mean square error of approximation (RMSEA), comparative fit index (CFI), Tucker-Lewis index (TLI), standardized root mean square residual (SRMR), Akaike information criterion (AIC), Bayesian information criterion (BIC), and sample-size adjusted Bayesian information criterion (SABIC). CFI compares fit to a null model where a value more than 0.90 is an acceptable fit [41]. TLI also does a comparison of fit to a null model while penalizing complexity, and here a value more than 0.90 is an acceptable fit. The SRMR value should be less than 0.08 for a good fit, and it measures average standardized residuals [42]. A value of 0.05–0.08 is an acceptable fit for RMSEA, while more than 0.10 represents Poor fit and measurement of fit per degree of freedom can be known using it [43]. Lastly, in the case of AIC, BIC, or SABIC, lower values indicate a better fit. The Kolmogorov–Smirnov test was used to test distributional assumptions, and the test shows normality after log transformation of the global score of PSQI and the scores of depression, anxiety, and stress.

Independent samples *t*-tests were conducted to compare PSQI and DASS-21 scores based on sex and institutional type. Chi-square tests were used to examine associations between categorical variables and sleep quality. Unadjusted and adjusted binary logistic regression was employed to identify predictors of poor sleep quality indicating crude odd ratio (COR) and adjusted odd ratio (AOR) with 95 % confidence interval. All analyses were carried out using SPSS version 25 and the “lavaan” package in R version 4.0.2.

### Ethical consideration

This study received approval (see Supplementary Material S2) from the higher study board of the Department of Population Science at Jatiya Kabi Kazi Nazrul Islam University (JKKNIU.PS.Ethical.2022.60), and all committee guidelines were diligently adhered to during the research.

## Results

A total of 378 students participated in the study. The mean age of the participants was 22.7 ± 3.2 years, with a mean body mass index (BMI) of 23.4 ± 3.6 kg/m^2^. On average, students reported spending 3.96 ± 2.67 h daily on social media or online gaming, while their average sleep duration was 6.0 ± 1.4 h per night. The mean global score on the PSQI was 7.7 ± 4.0, indicating poor average sleep quality. Scores from the DASS-21 revealed moderate levels of depression (mean 15.5 ± 11.4), anxiety (12.6 ± 9.1), and stress (16.6 ± 10.1) (Table 1).

**Table 1:** Means and standard deviations of the background characteristics of Bangladeshi medical students.

Factor/Variable	Mean (±SD)
Age (in years)	22.74 (±3.15)
BMI	23.37 (±3.56)
Daily time (in hours) spent on social media and online games	3.96 (±2.67)
Hours slept	6.02 (±1.38)
sleep quality	7.71 (±3.97)
Depression	15.52 (±11.40)
Anxiety	12.62 (±9.10)
Stress	16.64 (±10.12)

Abbreviations: BMI, body mass index; SD, standard deviation.

Overall, 67.2 % of students met the criteria for poor sleep quality. Additionally, 55.8 % of participants reported moderate to extremely severe depression, 58.7 % reported moderate to extremely severe anxiety, and 38.6 % reported moderate to extremely severe stress.

### Factorial validity

**Fig. 3:**
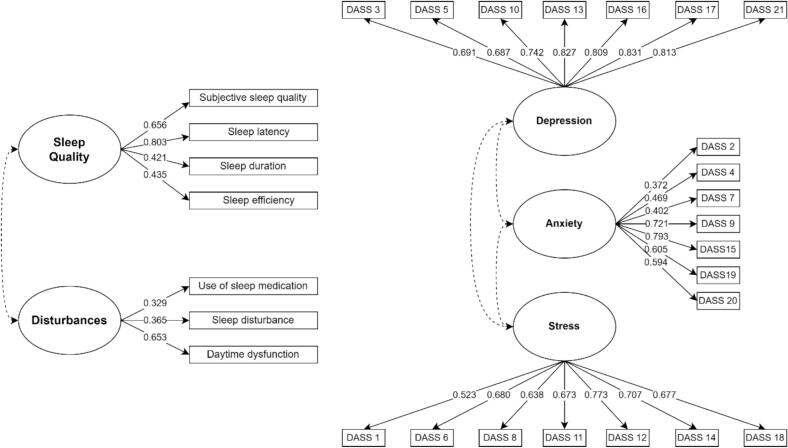
Item loadings from model 1 of PSQI (on the right) and model 1 of DASS-21 (on the left) Abbreviations: DASS, Depression, Anxiety, and Stress Scale.

The factorial validity of the PSQI was assessed using confirmatory factor analysis (CFA), as detailed in Table 2. In PSQI Model 1, all seven indicators were included, with the item loadings displayed in Fig. 3. In PSQI Model 2, two items, “use of sleep medication” and “sleep disturbance,” were excluded due to their factor loadings being below 0.40. PSQI Model 2 demonstrated an acceptable fit, with a CFI of 0.913 and an RMSEA of 0.098. Similarly, the factorial validity of the DASS-21 was evaluated through CFA, which compared three models. Item loadings of DASS Model 1 are shown in Fig. 3. In DASS Model 2, items with loadings below 0.40 were removed, and in Model 3, DASS item 7 was excluded. Model 3 achieved the best fit, with a CFI of 0.931 and a TLI of 0.921, both exceeding the 0.90 threshold for good model fit. The RMSEA was approximately 0.06, falling within the acceptable range [44,45]. The lower RMSEA, SRMR, and minimized AIC, BIC, and SABIC values confirmed DASS Model 3 as the most parsimonious and best-fitting structure.

**Table 2:** Analysis of Confirmatory Factor Analysis (CFA) Model of the Pittsburgh Sleep Quality Index (PSQI) and DASS-21 for the medical students in Bangladesh.

	Indicators	CFI	TLI	ACI	BIC	SABIC	RMSEA	SRMR	*p*-value
**PSQI**	**Model fit 1**	0.914	0.862	6276.670	6363.238	6293.436	0.101	0.046	<0.001
	Sleep Quality								
	Disturbances								
	**Model fit 2**	0.913	0.825	4758.719	4817.743	4770.151	0.098	0.048	<0.001
	Sleep Quality								
	Disturbances								
**DASS-21**	**Model fit 1**	0.910	0.898	18,890.682	19,150.385	18,940.982	0.072	0.046	<0.001
	Depression								
	Anxiety								
	Stress								
	**Model fit 2**	0.915	0.903	17,939.701	18,187.599	17,987.714	0.073	0.045	<0.001
	Depression								
	Anxiety								
	Stress								
	**Model fit 3**	0.931	0.921	16,959.439	17,195.532	17,005.166	0.068	0.039	<0.001
	Depression								
	Anxiety								
	Stress								

PSQI Model 1 represents the original model, including all seven components. PSQI Model 2 excludes the components “use of sleep medication” and “sleep disturbance” due to factor loadings (< 0.40).

DASS-21 Model 1 represents the initial model with all items included. DASS-21 Model 2 excludes DASS 2 because the factor loading was less than 0.40. DASS-21 Model 3 further excludes item 7 when it showed factor loading below 0.40 in Model 2.

Abbreviations: AIC, Akaike information criterion; BIC, Bayesian information criterion; CFA, Confirmatory factor analysis; CFI, Comparative fit index; RMSEA, Root mean square error of approximation; SABIC, Sample-size adjusted Bayesian information criterion; SRMR, Standardized root mean square residual; TLI, Tucker-Lewis index.

### Correlation patterns

**Fig. 4:**
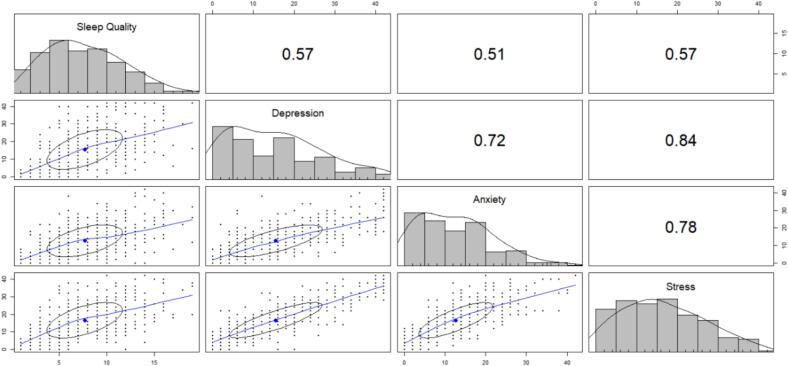
Scatter plot matrix for sleep quality and psychological distress among the medical students in Bangladesh

The scatter plot matrix in Fig. 4 reveals that poor sleep quality is moderately correlated with depression (r = 0.57, 95 % CI: 0.49–0.63), anxiety (*r* = 0.51, 95 % CI: 0.44–0.58), and stress (*r* = 0.57, 95 % CI: 0.50–0.64). Additionally, depression demonstrated a strong positive relationship with both anxiety (*r* = 0.72, 95 % CI: 0.67–0.77) and stress (*r* = 0.84, 95 % CI: 0.81–0.87). Furthermore, stress and anxiety were also strongly positively correlated (*r* = 0.78, 95 % CI: 0.73–0.81).

### Group differences

Independent samples *t*-tests (Table 3) were conducted to examine the effects of sex and medical type on sleep quality, depression, anxiety, and stress scores. The results indicate that male medical students reported better mental health and sleep quality compared to their female counterparts. Additionally, those from private medical institutions exhibited higher levels of depression and anxiety than students from government medical.

**Table 3:** Independent samples t-test of Bangladeshi medical students.

Test Variable	Grouping Variable		Mean	T value	df	p-value
PSQI	Sex	Male	6.40	−5.006	376	<0.001
		Female	8.32			
	Medical type	Government	7.14	−0.948	376	0.3
		Private	7.52			
Depression	Sex	Male	12.86	−4.446	376	<0.001
		Female	17.93			
	Medical type	Government	13.92	−2.667	376	0.008
		Private	17.05			
Anxiety	Sex	Male	11.22	−2.311	376	0.02
		Female	13.33			
	Medical type	Government	10.95	−3.305	376	0.001
		Private	13.97			
Stress	Sex	Male	14.10	−4.355	376	<0.001
		Female	18.49			
	Medical type	Government	15.32	−1.923	376	0.06
		Private	17.32			

Abbreviations: DASS, Depression, Anxiety, and Stress Scale.

Abbreviations: df, degrees of freedom.

### Factors associated with sleep quality

Table 4 presents the associations between sleep quality and various demographic, socio-economic, academic, and lifestyle factors among Bangladeshi medical students. Chi-square analyses revealed that poor sleep quality was associated with depression, anxiety, and stress. Furthermore, associations were found between sleep quality and sex, academic level, paternal occupation, family environment, screen time, smoking, career-building perceptions, perceived social value, and professional environment.

**Table 4:** Background characteristics and emotional distress wise distribution and prevalence of sleep quality and their association with sleep quality.

Factor	Category/Level	Total (%)	Good Sleep Quality (%)	Poor Sleep Quality (%)	p-value
Depression	No	167 (44.2)	95 (56.9)	72 (43.1)	<0.001
	Yes	211 (55.8)	29 (13.7)	182 (86.3)	
Anxiety	No	156 (41.3)	85 (54.5)	71 (45.5)	<0.001
	Yes	222 (58.7)	39 (17.6)	183 (82.4)	
Stress	No	232 (61.4)	111 (47.8)	121 (52.2)	<0.001
	Yes	146 (38.6)	13 (8.9)	133 (91.1)	
Body mass index (BMI)	Underweight	22 (5.8)	3 (13.6)	19 (86.4)	0.2
	Healthy weight	259 (68.5)	91 (35.1)	168 (64.9)	
	Overweight	81 (21.4)	26 (32.1)	55 (67.9)	
	Obesity	16 (4.2)	4 (25)	12 (75)	
Medical type	Government	220 (58.2)	77 (35)	143 (65)	0.3
	Private	158 (41.8)	47 (29.7)	111 (70.3)	
Current Professional	First professional	97 (25.7)	21 (21.6)	76 (78.4)	0.02
	Second professional	77 (20.4)	35 (45.5)	42 (54.5)	
	Third professional	90 (23.8)	33 (36.7)	57 (63.3)	
	Final professional	58 (15.3)	18 (31)	40 (69)	
	Post-graduation or equivalent	56 (14.8)	17 (30.4)	39 (69.6)	
Sex	Male	201 (53.2)	87 (43.3)	114 (56.7)	<0.001
	Female	177 (46.8)	37 (20.9)	140 (79.1)	
Religion	Islam	346 (91.8)	115 (33.2)	231 (66.8)	0.7
	Hindu	29 (7.7)	8 (27.6)	21 (72.4)	
	Others	2 (0.5)	1 (50)	1 (50)	
Permanent residence	Rural	179 (47.4)	67 (37.4)	112 (62.6)	0.07
	Urban	199 (52.6)	57 (28.6)	142 (71.4)	
Current residence	Hall	132 (34.9)	51 (38.6)	81 (61.4)	0.1
	Rented house or mess	104 (27.5)	34 (32.7)	70 (67.3)	
	Own house	142 (37.6)	39 (27.5)	103 (72.5)	
Family Type	Nuclear	310 (82)	108 (34.8)	202 (65.2)	0.07
	Joint	68 (18)	16 (23.5)	52 (76.5)	
Number of siblings	0–3	306 (81)	96 (31.4)	210 (68.6)	0.2
	4–7	72 (19)	28 (38.9)	44 (61.1)	
Father's education	Illiterate/Primary	16 (4.2)	3 (18.8)	13 (81.3)	0.05
	Secondary	42 (11.1)	8(19)	34 (81)	
	Higher secondary or above	320 (84.7)	113 (35.3)	207 (64.7)	
Father's occupation	Service holder	162 (42.9)	44 (27.2)	118 (72.8)	0.01
	Business	112 (29.6)	34 (30.4)	78 (69.6)	
	Farmer or others	104 (27.5)	46 (44.2)	58 (55.8)	
Mother's education	Illiterate/Primary	33 (8.7)	10 (30.3)	23 (69.7)	0.2
	Secondary	95 (25.1)	25 (26.3)	70 (73.7)	
	Higher secondary or above	250 (66.1)	89 (35.6)	161 (64.4)	
Mother's occupation	Housewife	276 (73.0)	83 (30.1)	193 (69.9)	0.08
	Service holder	69 (18.3)	25 (36.2)	44 (63.8)	
	Others	33 (8.7)	16 (48.5)	17 (51.5)	
Friendly family environment	No	44 (11.6)	4 (9.1)	40 (90.9)	<0.001
	Neutral	95 (25.1)	26 (27.4)	69 (72.6)	
	Yes	239 (63.2)	94 (39.3)	145 (60.7)	
Daily average study hour	1–3	196 (51.9)	67 (34.2)	129 (65.8)	0.3
	4–6	145 (38.4)	49 (33.8)	96 (66.2)	
	6+	37 (9.8)	8 (21.6)	29 (78.4)	
Result	Fail	33 (8.7)	6 (18.2)	33 (81.8)	0.06
	Pass	345 (91.3)	118 (34.2)	227 (65.8)	
Daily time spent on social media and online games	0–3 Hours	211 (55.8)	86 (40.8)	125 (59.2)	<0.001
	3–6 Hours	118 (31.2)	33 (28)	85 (72)	
	6+ Hours	49 (13)	5 (10.2)	44 (89.8)	
Relationship status	Single or Unmarried	281 (74.3)	97 (34.5)	184 (65.5)	0.2
	Married	54 (14.3)	18 (33.3)	36 (66.7)	
	Separated/Divorced/ Wedded/Engaged	43 (11.4)	9 (20.9)	34 (79.1)	
Smoking status	No	304 (80.4)	92 (30.3)	212 (69.7)	0.03
	Yes	74 (19.6)	32 (43.2)	42 (56.8)	
Alcohol	No	342 (90.5)	111 (32.5)	231 (67.5)	0.7
	Yes	36 (9.5)	13 (36.1)	23 (63.9)	
Night duty and sleep disturbance	No	68 (18)	21 (30.9)	41 (69.1)	0.7
	Yes	310 (82)	103 (33.2)	207 (66.8)	
Satisfied with the initiatives by govt.	No	228 (60.3)	73 (32)	155 (68)	0.7
	Yes	150 (39.7)	51 (34)	99 (66)	
Career-building opportunity	No	194 (51.3)	44 (22.7)	150 (77.3)	<0.001
	Neutral	100 (26.5)	49 (49)	51 (51)	
	Yes	84 (22.2)	31 (36.9)	53 (63.1)	
Social value	No	213 (56.3)	55 (25.8)	158 (74.2)	0.004
	Neutral	80 (21.2)	34 (42.5)	46 (57.5)	
	Yes	85 (22.5)	35 (41.2)	50 (58.8)	
Professional environment	No	269 (71.2)	74 (27.5)	195 (72.5)	0.002
	Neutral	67 (17.7)	32 (47.8)	35(52.2)	
	Yes	42 (11.1)	18 (42.9)	24 (57.1)	

### Predictors of poor sleep quality

Table 5 represents the Impact of emotional distress and background characteristics on sleep quality. Binary logistic regression identified that depression (AOR = 2.61, 95 % CI: 1.37–4.99), anxiety (AOR = 1.88, 95 % CI: 1.01–3.52), and stress (AOR = 2.77, 95 % CI: 1.25–6.14) increased the odds of poor sleep. Male students were less than half as likely as female students to experience poor sleep (AOR = 0.42, 95 % CI: 0.25–0.73). A clear dose–response relationship was observed for screen time: students spending more than six hours daily on social media or gaming had six times the odds of experiencing poor sleep (AOR = 6.05, 95 % CI: 2.31–15.89). Smoking showed an inverse association with poor sleep (AOR = 0.54, 95 % CI: 0.31–0.94); however, this result may be affected by unmeasured confounding factors. Students with negative perceptions of career-building opportunities were nearly three times more likely to experience poor sleep quality (AOR = 0.38, 95 % CI: 0.22–0.66), demonstrating the joint influence of psychosocial and behavioral factors on sleep health among medical students. Apart from these, unadjusted logistic regression shows all the eleven variables tested except the current professional of the medical student have an effect on sleep quality.

**Table 5:** Impact of emotional distress and background characteristics on sleep quality by binary logistic regression analysis.

Variable	Category	Unadjusted Logistic Regression		Adjusted Logistic Regression	
		COR (95 % CI)	p-value	AOR (95 % CI)	p-value
Depression	Yes	1.13 (1.08, 1.22)	<0.001	2.61 (1.37, 4.99)	0.004
	No	Reference			
Anxiety	Yes	1.19 (1.12, 1.33)	<0.001	1.88 (1.01, 3.52)	0.05
	No	Reference			
Stress	Yes	1.12 (1.06, 1.22)	<0.001	2.77 (1.25, 6.14)	0.01
	No	Reference			
Sex	Male	0.35 (0.22, 0.55)	<0.001	0.42 (0.25, 0.73)	0.002
	Female	Reference			
Current Professional	First professional	1.57 (0.75, 3.33)	0.2	1.14 (0.48, 2.71)	0.8
	Second professional	0.52 (0.25, 1.08)	0.08	0.53 (0.23, 1.26)	0.2
	Third professional	0.75 (0.36, 1.54)	0.4	0.71 (0.31, 1.64)	0.4
	Final professional	0.97 (0.43, 2.14)	0.9	0.76 (0.30, 1.92)	0.6
	Post-graduation or equivalent	Reference			
Father's occupation	Business	2.12 (1.26, 3.57)	0.004	1.04 (0.54, 2.01)	0.9
	Farmer or others	1.81 (1.04, 3.18)	0.04	0.78 (0.42, 1.46)	0.4
	Service holder	Reference			
Friendly family environment	Neutral	1.86 (0.81, 2.92)	0.001	0.47 (0.14, 1.64)	0.2
	Yes	0.54 (0.02, 1.06)	0.04	0.34 (0.11, 1.07)	0.07
	No	Reference			
Daily time spent on social media and online games	3–6 Hours	1.18 (1.06, 1.54)	<0.001	1.77 (1.09, 2.88)	0.02
	6+ Hours	1.35 (1.11, 2.23)	0.02	6.05 (2.31, 15.89)	<0.001
	0–3 Hours	Reference			
Smoking status	Yes	0.56 (0.04, 1.08)	0.03	0.54 (0.31, 0.94)	0.03
	No	Reference			
Career-building opportunity	Neutral	0.69 (0.13, 1.24)	0.02	0.38 (0.22, 0.66)	0.001
	Yes	0.61 (0.34, 1.10)	0.1	0.66 (0.35, 1.26)	0.2
	No	Reference			
Social value	Neutral	0.69 (0.17, 1.23)	0.01	0.72 (0.39, 1.34)	0.3
	Yes	0.94 (0.51, 1.76)	0.9	0.62 (0.32, 1.18)	0.1
	No	Reference			
Professional environment	Neutral	0.68 (0.01, 1.34)	0.05	0.63 (0.34, 1.16)	0.1
	Yes	0.82 (0.38, 1.78)	0.6	0.79 (0.36, 1.72)	0.5
	No	Reference			

CI: Confidence Interval, AOR: Adjusted Odds Ratio, COR: Crude Odds Ratio

## Discussions

This study revealed a high prevalence of poor sleep quality and psychological distress among Bangladeshi medical students, higher than the rates reported for other university students [12,13,16] and general youth populations in Bangladesh [14,15]. Similar findings were observed among Swiss medical students, who reported higher rates of insomnia compared to general students or emergency personnel [10], while students in several South Asian countries reported fewer sleep-related problems [18]. On a global scale, the prevalence in this study exceeds estimates from Southeast Asia [46] and other regions [47]. Comparable outcomes have been documented in Iran [48], Indonesia [28], India [29], and Egypt [49], whereas European students generally report better sleep, with the exception of students in Croatia [11]. Interestingly, Bangladeshi students exhibited better sleep quality than those in Brazil [50]. This geographical pattern suggests that poorer sleep is more prevalent in the Global South, where academic and socioeconomic stressors are similar, while students in the Global North tend to experience healthier sleep. Factors such as Bangladesh's high population density [51], an overburdened health system, and recent disruptions from the pandemic likely contribute to the heightened stress and impaired sleep among medical trainees. Consistent with international research, the COVID-19 pandemic has exacerbated psychological distress, particularly among students with prior mental health issues [23]. Given the emotional and cognitive demands of medical education [[1], [2], [3], [4], [5]], these findings help explain the elevated levels of anxiety, depression, suicidality, and poor sleep quality reported in other studies [6,7,10,52,53].

Depression levels in this study were comparable to earlier estimates in Bangladesh [27], but were higher than those found in India [37], Iran [48], Jordan [54], and Croatia [11], and even surpassed the levels reported by Ukrainian students in conflict-free regions [55], indicating noteworthy systemic and academic pressures. The prevalence of anxiety was approximately 1.5 times greater than in similar studies from Jordan [54], India [37], and Iran [48]. Meanwhile, stress levels were consistent with previous Bangladeshi data [27]. Although stress has decreased by about 16 % over the past decade [21], it remains higher than in Croatia [11] and lower than in the Middle East [33,54]. Australian students reported lower levels of distress [56], while Brazilian [23] and Pakistani [57] students exhibited higher and lower levels, respectively. Collectively, these findings confirm that medical students, particularly in developing countries, experience greater mental health burdens than the general youth population [32]. The mean screen exposure of nearly four hours daily aligns with findings from Indonesia [26], whereas the average sleep duration of six hours is consistent with results from Bangladesh [22] and Saudi Arabia [33]. Confirmatory factor analysis validated a two-factor model for the PSQI and a three-factor model for the DASS-21, in line with prior validation studies in Iran [34], Spain [44], Sri Lanka [45], Ethiopia [58], and Portugal [59], thereby confirming the reliability of these instruments.

Sex and institutional differences were substantial. Male students reported better sleep and lower distress, which aligns with studies from Bangladesh [12], Egypt [49], Sweden [10], and Croatia [11], although this contrasts with findings from India [29]. Across various contexts [11,37,55,56], female students appear to be more psychologically vulnerable, potentially due to biological sex and social pressures. Institutional differences were also apparent: while earlier research from Bangladesh indicated higher stress levels in public institutions [21], this study found greater depression and anxiety among private medical students, likely reflecting financial strain, curriculum structure, or social dynamics. Depression emerged as the strongest predictor of poor sleep, with affected students more than twice as likely to report inadequate rest, echoing findings from Croatia [11] and Switzerland [10]. Anxiety and stress were also significant predictors, consistent with results from Ukraine [60] and Saudi Arabia [33]. The inverse relationship between being male and poor sleep mirrors studies from Bangladesh [12], Egypt [36], and Switzerland [10]. Prolonged screen time demonstrated a clear dose–response relationship with poor sleep: three to six hours of use increased the risk by 50 %, while over six hours raised it six times, consistent with evidence from Bangladesh [14,16] and Indonesia [28].

An unexpected finding was that smokers reported better sleep quality, which contradicts prior literature, including Polish research showing the opposite trend [60]. This discrepancy likely reflects reporting bias or unmeasured confounding, as nicotine dependence can create a false sense of relaxation while disrupting sleep physiology. Students who were pessimistic about their career prospects were nearly three times more likely to experience poor sleep, highlighting the impact of perceived job insecurity and uncertainty about future employment on mental well-being. These psychosocial factors, combined with heavy academic workloads, limited recreational opportunities, and excessive digital use, appear to be central to the interaction between sleep and mental health among Bangladeshi medical students.

This study has several limitations. Its cross-sectional design restricts causal inference, and reliance on self-reporting may introduce recall or social desirability bias. Specifically, reverse causality cannot be excluded, as inadequate sleep quality or psychological distress may result in heightened social media usage and online gaming, rather than these activities inducing poor sleep. Temporal direction could not be determined because exposure data was gathered at the time of the survey. Future research should employ longitudinal designs to assess temporal relationships, integrate objective sleep-tracking data, and explore institutional or cultural moderators. Some students may have underreported symptoms due to stigma, while survey fatigue could have affected accuracy. Besides, smoking status was recorded as a binary variable in this study, which did not account for former smokers, the intensity of tobacco use, and alternative nicotine sources (e.g., e-cigarettes). As nicotine significantly impacts sleep quality, lack of more relevant questions may limit the depth of the analysis in this specific domain. However, the use of validated instruments, a large multi-institutional sample, and robust analytical methods enhance confidence in the findings. Mixed-method studies could also provide deeper insights into the contextual mechanisms linking psychosocial stress, digital behavior, and sleep outcomes among medical students in low-resource environments.

## Conclusion

This study identified several factors that influence sleep quality among Bangladeshi medical students. Depression, anxiety, stress, and the extended time spent daily on social media and online gaming were strongly associated with poorer sleep quality. In contrast, being male, smoking, and perceiving career-building opportunities were linked to better sleep quality. The unexpected positive association between smoking and sleep may reflect coping strategies, reporting bias, or unmeasured confounders, and thus requires cautious interpretation. The prevalence of poor sleep quality was notably high, and additional factors including academic year, father's occupation, family support, social value, and professional environment were also associated with sleep quality.

The findings emphasize the need for targeted interventions at both institutional and policy levels, especially in the areas of stress management and the promotion of sleep hygiene. Special attention should be given to female students, who tend to experience poorer sleep. Authorities must also address mental health challenges, especially among students in private medical colleges. Future research should investigate the long-term consequences of inadequate sleep through longitudinal studies, evaluate the effectiveness of these interventions, and utilize qualitative methods to gain a deeper understanding of students' lived experiences. This includes exploring factors that contribute to sleep disparities among female students and the higher levels of psychological distress observed in private institutions.

The following is the supplementary data related to this article.

Supplementary Material S1 Supplementary Material S1

Supplementary Material S2 Supplementary Material S2

## CRediT authorship contribution statement

**Abdul Muyeed:** Writing – review & editing, Writing – original draft, Visualization, Validation, Supervision, Methodology, Investigation, Formal analysis, Data curation, Conceptualization. **Ratul Rahman:** Writing – review & editing, Writing – original draft, Visualization, Validation, Methodology, Formal analysis, Data curation. **Sumaiya Islam Suchi:** Writing – review & editing, Writing – original draft, Visualization, Validation, Methodology, Data curation. **Kawsar Ahmed:** Writing – review & editing, Writing – original draft, Visualization, Validation, Data curation. **Tahmina Akter Tithi:** Writing – review & editing, Writing – original draft, Visualization, Validation, Data curation.

## Funding

This research did not receive any specific grant from funding agencies in the public, commercial, or not-for-profit sectors.

## Declaration of competing interest

The authors ensure no conflicts of interest or personal relationships that could have appeared to influence the work reported in this paper.

## References

[1] Arif N.M.N.A., Roslan N.S., Ismail S.B., Nayak R.D., Jamian M.R., Mohamad Ali Roshidi A.S. (2021). Prevalence and associated factors of psychological distress and burnout among medical students: findings from two campuses. Int J Environ Res Public Health.

[2] Dyrbye L.N., Thomas M.R., Shanafelt T.D. (2006). Systematic review of depression, anxiety, and other indicators of psychological distress among U.S. and Canadian medical students. Acad Med.

[3] Jeppu Ashok Kumar, Ferdous Azam S.M., Kumar Kavitha Ashok (2022). Comparing the psychological distress among the medical students at different levels of training. Indian J Public Health Res Dev.

[4] Langness S., Rajapuram N., Marshall M., Rahman A.S., Sammann A. (2022). Risk factors associated with student distress in medical school: associations with faculty support and availability of wellbeing resources. PloS One.

[5] Rubaba Azim S., Kalinin V., Hocaoglu C., Mohamed S. (2021). Anxiety Disord. - New Achiev.

[6] Resident in Saudi Board of Preventive Medicine, A A, M. A, Resident in Saudi Board of Preventive Medicine, A E, Resident in Saudi Board of Preventive Medicine (2023). Factor associated with stress, anxiety, and depression among medical students in Tabuk city, 2022. Int J Adv Res.

[7] Maser B., Danilewitz M., Guérin E., Findlay L., Frank E. (2019). Medical student psychological distress and mental illness relative to the general population: A Canadian cross-sectional survey. Acad Med.

[8] An Y., Ji X., Zhou L., Liu J. (2023). Sleep and subjective well-being among chinese adolescents: resilience as a mediator. Asian J Soc Health Behav.

[9] Hapsari E.A., Rohmatullayaly E.N., Widayati K.A. (2024). Technostress and sleep quality among university students in Indonesia: A cross-sectional study. Asian J Soc Health Behav.

[10] Regli J., Sadeghi-Bahmani D., Rigotti V., Stanga Z., Ülgür I.I., Fichter C. (2024). Psychiatric characteristics, symptoms of insomnia and depression, emotion regulation, and social activity among Swiss medical students. J Clin Med.

[11] Vidović S., Rakić N., Kraštek S., Pešikan A., Degmečić D., Zibar L. (2025). Sleep quality and mental health among medical students: A cross-sectional study. J Clin Med.

[12] Al-Mamun F., Mamun M., Hasan M.E., MM Almerab, Gozal D. (2024). Exploring sleep duration and insomnia among prospective university students: a study with geographical data and machine learning techniques. Nat Sci Sleep.

[13] Banna MdHA, Hamiduzzaman M., Akter S., Seidu A., Begum A., Yeasmin N. (2025). University students’ sociodemographics, physical inactivity, and inadequate and poor-quality sleep are associated with their overweight/obesity: findings From a Case–Control Study in Bangladesh. Health Sci Rep.

[14] Islam MdR, Ahmed O., Naher L., Islam MdN. (2025). The association between problematic smartphone use and subjective well-being in Bangladeshi youths: mediating role of sleep quality. Addict Behav Rep.

[15] Muyeed A., Talukder A., Rahman R., Rumi M.H. (2025). Assessing the impact of emotional distress on internet gaming disorder among youth in Bangladesh: a cross-sectional survey. Ment Health Soc Incl.

[16] Nurunnabi M., Khan M., Kaiser F., Abbas M., Tabassum T., Farha T. (2025).

[17] Sultana S., Chowdhury M.S., Akbar F.M., Shams N.A.B. (2022). Sleep patterns among the pre-clinical medical students of a selected medical College in Bangladesh. J Armed Forces Med Coll Bangladesh.

[18] Chowdhury A.I., Ghosh S., Hasan M.F., Siam K.K.A., Azad F. (2020). Prevalence of Insomnia among University Students in South Asian Region: a systematic review of studies. J Prev Med Hyg.

[19] Hasan M.T., Hossain S., Gupta R.D., Podder V., Mowri N.A., Ghosh A. (2022). Depression, sleeping pattern, and suicidal ideation among medical students in Bangladesh: a cross-sectional pilot study. J Public Health.

[20] Mahmud S., Mohsin M., Muyeed A., Nazneen S., Abu Sayed Md, Murshed N. (2023). Machine learning approaches for predicting suicidal behaviors among university students in Bangladesh during the COVID-19 pandemic: A cross-sectional study. Medicine (Baltimore).

[21] Eva E.O., Islam M.Z., Mosaddek A.S.M., Rahman M.F., Rozario R.J., Iftekhar A.F.M.H. (2015). Prevalence of stress among medical students: a comparative study between public and private medical schools in Bangladesh. BMC Res Notes.

[22] IMAA Khaliq, Abdullah Z.N., Abdullah J.A., Mohammed Z.A. (2023). Prevalence of stress and sleep disorders among medical students at Al-Kindy College of Medicine and its impact on academic performance. South Asian Res J Appl Med Sci.

[23] Alves R., Aguilar-da-Silva R., Vane L., Vieira J., Vane M., Will E. (2022).

[24] Matheson K.M., Barrett T., Landine J., McLuckie A., Soh N.L.-W., Walter G. (2016). Experiences of psychological distress and sources of stress and support during medical training: a survey of medical students. Acad Psychiatry.

[25] Jahrami H., AlKaabi J., Trabelsi K., Pandi-Perumal S.R., Saif Z., Seeman M.V. (2023). The worldwide prevalence of self-reported psychological and behavioral symptoms in medical students: An umbrella review and meta-analysis of meta-analyses. J Psychosom Res.

[26] Mahmud S., Hossain S., Muyeed A., Islam M.M., Mohsin Md (2021). The global prevalence of depression, anxiety, stress, and, insomnia and its changes among health professionals during COVID-19 pandemic: A rapid systematic review and meta-analysis. Heliyon.

[27] Lama S.M., Ahad MdTE (2023).

[28] Alya Farhana H., Rahmatul Islamiyah W., Irwadi I., Dr Fidiana (2022). Relationship between smartphone screen time and sleep quality (PSQI) on preclinical medical students of Airlangga University, Surabaya. Int J Res Publ.

[29] Lohitashwa R., Kadli N., Kisan R., S A., Deshpande D. (2015). Effect of stress on sleep quality in young adult medical students: a cross sectional study. Int J Res Med Sci.

[30] Ahmed S. (2024).

[31] Tajmim T. (2023).

[32] Pagnin D., De Queiroz V. (2015). Comparison of quality of life between medical students and young general populations. Educ Health.

[33] Almojali A.I., Almalki S.A., Alothman A.S., Masuadi E.M., Alaqeel M.K. (2017). The prevalence and association of stress with sleep quality among medical students. J Epidemiol Glob Health.

[34] Shadzi M.R., Rahmanian M., Heydari A., Salehi A. (2024). Structural validity of the Pittsburgh sleep quality index among medical students in Iran. Sci Rep.

[35] Mahmood T., Afroz R., Chowdhury J.P., Khan F., Zaman M.S., Khan M.A.S. (2025). Cross-cultural adaptation and psychometric evaluation of Pittsburgh sleep quality index Bangla (PSQI-BN) in Bangladeshi population. Sleep Biol Rhythms.

[36] Buysse D.J., Reynolds C.F., Monk T.H., Berman S.R., Kupfer D.J. (1989). The Pittsburgh sleep quality index: A new instrument for psychiatric practice and research. Psychiatry Res.

[37] Gupta S., Potdar P., Singh R., Shukla K.K. (2022). A cross sectional study on depression, anxiety and stress among the first-year medical students. Int J Commun Med Public Health.

[38] Alim S.A.H.M., Kibria S.M.E., Lslam M.J., Uddin M.Z., Nessa M., Wahab M.A. (2017). Translation of DASS 21 into Bangla and validation among medical students. Bangladesh J Psychiatry.

[39] Mamun M.A., Hossain MdS, Griffiths M.D. (2022). Mental health problems and associated predictors among Bangladeshi students. Int J Ment Heal Addict.

[40] Lovibond P.F., Lovibond S.H. (2015).

[41] Bentler P.M. (1990). Comparative fit indexes in structural models. Psychol Bull.

[42] Hu L., Bentler P.M. (1999). Cutoff criteria for fit indexes in covariance structure analysis: conventional criteria versus new alternatives. Struct Equ Model Multidiscip J.

[43] Browne M.W., Cudeck R. (1992). Alternative ways of assessing model fit. Sociol Methods Res.

[44] Otero P., Simón M.A., Bueno A.M., Blanco V., Vázquez F.L. (2022). Factorial structure and psychometric properties of the Spanish version of the Pittsburgh sleep quality index in non-professional caregivers. Healthcare.

[45] Teh C.K., Ngo C.W., Zulkifli R.A.B., Vellasamy R., Suresh K. (2015). Depression, anxiety and stress among undergraduate students: A cross sectional study. Open J Epidemiol.

[46] Ferry D.S., Sharon L.H., Tahereh S.I., Winny N.W., Mohamad H.H. (2024). Prevalence of poor sleep quality based on Pittsburgh sleep quality index (PSQI) among medical students in Southeast Asia: a systematic review and meta-analysis. J Sleep Disord Manag.

[47] Binjabr M.A., Alalawi I.S., Alzahrani R.A., Albalawi O.S., Hamzah R.H., Ibrahim Y.S. (2023). The worldwide prevalence of sleep problems among medical students by problem, country, and COVID-19 status: a systematic review, meta-analysis, and Meta-regression of 109 studies involving 59427 participants. Curr Sleep Med Rep.

[48] Shafiee A., Teymouri Athar M.M., Seighali N., Amini M.J., Hajishah H., Arabazadeh Bahri R. (2024). The prevalence of depression, anxiety, and sleep disturbances among medical students and resident physicians in Iran: a systematic review and meta-analysis. PloS One.

[49] Ahmed Salama A. (2017). Sleep quality in medical students, Menoufia university, Egypt. Egypt Fam Med J.

[50] Alves S.F.L., Santos T.A.B.P.D.S., Da Silva M.L., Cunha K.D.C. (2025). Heart rate variability, sleep quality and physical activity in medical students. Sleep Epidemiol.

[51] United Nations Population Fund (UNFPA) Bangladesh (2025).

[52] Mohamed Musa S.A., Mohamed A.M., Osman S.M. (2023). Psychological distress and suicidal behavior among medical students at Khartoum state universities, 2021-2022. Int J Med Stud.

[53] Nair M., Moss N., Bashir A., Garate D., Thomas D., Fu S. (2023). Mental health trends among medical students. Baylor Univ Med Cent Proc.

[54] Dodin Y., Obeidat N., Dodein R., Seetan K., Alajjawe S., Awwad M. (2024). Mental health and lifestyle-related behaviors in medical students in a Jordanian university, and variations by clerkship status. BMC Med Educ.

[55] Gusakova I.V., Konovalov S.V., Hmel L.L. (2023). Depression, anxiety and stress (according to DASS-21 test results) among students of Vinnytsya national medical university. Rep Vinnytsia Natl Med Univ.

[56] Bore M., Kelly B., Nair B. (2016). Potential predictors of psychological distress and well-being in medical students: a cross-sectional pilot study. Adv Med Educ Pract.

[57] Khalid S., Hotiana U.A., Muzzamil Z., Mumtaz S., Akram S. (2022). Psychological distress in medical students: cross sectional study. Pak J Med Health Sci.

[58] Tsegaye B.S., Asemu M.M., Hailu H.B. (2024). Construct validity and reliability of Amharic version of DASS-21 scale among Ethiopian Defense University College of Health Science students. BMC Health Serv Res.

[59] Laranjeira C., Querido A., Sousa P., Dixe M.A. (2023). Assessment and psychometric properties of the 21-item depression anxiety stress scale (DASS-21) among Portuguese higher education students during the COVID-19 pandemic. Eur J Investig Health Psychol Educ.

[60] Dąbrowska-Galas M., Ptaszkowski K., Dąbrowska J. (2021). Physical activity level, insomnia and related impact in medical students in Poland. Int J Environ Res Public Health.

