# Supplementary material for: Sleep quality and psychological distress among Bangladeshi medical students: Prevalence, predictors, and sex-institutional differences
Source: Glob Epidemiol. 2026 Jan 6;11:100243. doi: 10.1016/j.gloepi.2026.100243 (PMC12818157; doi:10.1016/j.gloepi.2026.100243)
Supplement: Supplementary Material S1 [file mmc1.docx]

**Prevalence and Risk Factors of Depression, Anxiety, Stress and Insomnia among Medical Students in Bangladesh: A Cross-sectional Survey**

N.B. All the information will be strictly used for research purpose. The information will not be disclosed to others.

Instruction: Please write down the information when necessary and when needed put a tick (√) on your desired answer.

Dear participants,

The major aim of this research is to estimate the prevalence of depression, anxiety, stress and insomnia among the medical students in Bangladesh. Also aim to figure out the influencing factors that are associated with the depression, anxiety, stress and insomnia of medical students. Hopefully this research will recommend medical college authorities and policy makers to take necessary initiatives for addressing these influencing factors to improve the mental health condition of medical students.

If you have any query or face any difficulties in completing this survey then please feel free to contact me.

Regards;  
Abdul Muyeed                                                                                                                      
Assistant Professor                                                                                                            
Department of Statistics,                                                                                                  
Jatiya Kabi Kazi Nazrul Islam University (JKKNIU), Bangladesh                            
Email: [amuyeed@isrt.ac.bd](mailto:amuyeed@isrt.ac.bd)                                                                                           
Mobile:+88 01725912378
         &
Dr. Kawsar Ahmed        
MBBS, DMRD (Part 2)    
Birdem General Hospital  
Email: [drkawsar.kmc@gmail.com](mailto:drkawsar.kmc@gmail.com)   
Mobile: +88 01736921661

Are you willing to participate in this survey?

- Yes
- No

**Respondent's background information**

**Socio-demographic and socio-economic information**

Type of your medical college

- Government
- Private

Current Prof (Professional)

- 1st
- 2nd
- 3rd
- 4th
- Post graduation or equivalent

Age in years_______________________________

Height in inches ((1 feet = 12 inches))_______________________________

Weight in kg_______________________________

Gender

- Male
- Female
- Others

Religion

- Islam
- Hindu
- Others

Origin/ Permanent residence

- Rural
- Urban

Current residence

- Hall
- Rent house or Mess
- Own house

Family type

- Nuclear
- Joint or Extended

Number of siblings_______________________________

Father’s Education

- Illiterate
- Primary
- Secondary
- Higher secondary
- Above

Father’s Occupation

- Service holder
- Businessman
- Farmar
- Others

Mother's education

- Illiterate
- Primary
- Secondary
- Higher secondary
- Above

Mother’s Occupation

- House wife
- Service holder
- Others

Family income (monthly) in taka_______________________________

Do you think your family environment is friendly?

- Strongly disagree
- Disagree
- Neutral
- Agree
- Strongly agree

Daily average study hour

- 1-3 hours
- 4-6 hours
- 7-9 hours
- ≥9 hours

Result (last professional)

- Pass
- Fail

Relationship status

- Single
- Married
- Engaged

How much daily average time (in hours) do you spend in social media (Facebook, Instagram, Twitter, and Others) and online games?_______________________________

Smoking status

- Yes
- No

Did you take alcohol or any other drugs in last three (3) months?

- Yes
- No

How many days do you have night duty in a week? _______________________________

Do you think night duty causes sleep disturbance?

- Yes
- No

Did you or your family member(s) or relatives get infected by novel coronavirus (COVID-19) during this pandemic?

- Yes
- No

Are you satisfied with the initiatives (safety equipment, vaccination, exam policy during pandemic) taken by government for medical students and practitioners at the time of COVID-19 pandemic?

- Yes
- No

Do you feel that in aspect of career building your profession has adequate opportunity in our country?

- Strongly disagree
- Disagree
- Neutral
- Agree
- Strongly agree

Do you think your profession get enough social value in our country?

- Strongly disagree
- Disagree
- Neutral
- Agree
- Strongly agree

Do you think you have good professional environment in our country?

- Strongly disagree
- Disagree
- Neutral
- Agree
- Strongly agree

**Depression, Anxiety and Stress Scale (DASS)**

Instructions: Please read each statement and circle a number 0, 1, 2 or 3 which indicates how much the statement applied to you OVER THE PAST WEEK. There are no right or wrong answer. The rating scale is as follows:

0 :  Did not apply to me at all
1 :  Applied to me to some degree, or some of the time
2 :  Applied to me to a considerable degree or a good part of time
3 :  Applied to me very much or most of the time

Please mark your condition

|  | 0 | 1 | 2 | 3 |
| --- | --- | --- | --- | --- |
| (1) I found it hard to wind down (s)-উত্তেজনা প্রশমিত করা আমার জন্য কঠিণ ছিলো |  |  |  |  |
| (2) I was aware of dryness of my mouth (a)-আমি আমার মুখের শুষ্কতা সম্পর্কে সচেতন ছিলাম |  |  |  |  |
| (3) I couldn’t seem to experience any positive feeling at all(d)-আমি মোটেও কোনও ইতিবাচক অনুভূতি অনুভব করতে পারিনি |  |  |  |  |
| (4) I experienced breathing difficulty (e.g. excessively rapid breathing, breathlessness in the absence of physical exertion)(a)- আমার শ্বাসকষ্টের অভিজ্ঞতা হয়েছে (যেমন অত্যধিক দ্রুত শ্বাস প্রশ্বাস, শারীরিক পরিশ্রম ছাড়াই শ্বাসকষ্ট ইত্যাদি) |  |  |  |  |
| (5) I found it difficult to work up the initiative to do things(d)-কোনকিছু শুরু করার উদ্যোগ নিতেই আমার সমস্যা হচ্ছিলো |  |  |  |  |
| (6) I tended to over-react to situations(s)-কোন পরিস্থিতিতে অতিরিক্ত প্রতিক্রিয়া দেখানোর প্রবণতা বেড়েছে |  |  |  |  |
| (7) I experienced trembling (e.g. in the hands)(a)-আমার কাপুনির (যেমনঃ হাতে) অভিজ্ঞতা হয়েছে |  |  |  |  |
| (8) I felt that I was using a lot of nervous energy(s)-আমি অনুভব করেছি যে আমি প্রচুর স্নায়বিক শক্তি ব্যবহার করছি |  |  |  |  |
| (9) I was worried about situations in which I might panic and make a fool of myself(a)-আমি উদ্বিগ্ন ছিলাম এমন পরিস্থিতির জন্য যেখানে আমি আতঙ্কিত হয়ে নিজেকে বোকা বানাই |  |  |  |  |
| (10) I felt that I had nothing to look forward to(d)-আমি অনুভব করেছি যে আমার জন্য আশানুরুপ কিছু অবশিষ্ট নেই |  |  |  |  |
| (11) I found myself getting agitated(s)-আমি প্রাঃয়শই বিরক্ত থাকি |  |  |  |  |
| (12) I found it difficult to relax(s)-আমার রিল্যাক্স থাকতে সমস্যা হয় |  |  |  |  |
| (13) I felt downhearted and blue(d)-আমি নিস্তেজ ও হতাশ অনুভব করেছি |  |  |  |  |
| (14) I was intolerant of anything that kept me from getting on with what I was doing(s)-আমি সবকিছুতেই অসহিষ্ণু ছিলাম যা আমাকে যা করছিলাম তা থেকে বিরত রেখেছে |  |  |  |  |
| (15) I felt I was close to panic(a)-আমার মনে হয়েছিল আমি প্রায় পেনিকড হয়ে গেছি |  |  |  |  |
| (16) I was unable to become enthusiastic about anything(d)-আমি কোনও বিষয়েই উৎসাহী হতে পারিনি |  |  |  |  |
| (17) I felt I wasn’t worth much as a person(d)-আমার মনে হয়েছে একজন ব্যক্তি হিসাবে আমি মূল্যহীন |  |  |  |  |
| (18) I felt that I was rather touchy(s)-আমি অনুভব করেছি যে আমি স্পর্শকাতর |  |  |  |  |
| (19) I was aware of the action of my heart in the absence of physical exertion (e.g. sense of heart rate increase, heart missing a beat)(a)-আমি শারীরিক পরিশ্রম না করলেও আমার হার্টের ক্রিয়া সম্পর্কে সচেতন ছিলাম (উদাঃ হৃদস্পন্দনের হার বাড়ার অনুভূতি, হার্টের অনুপস্থিতি ইত্যাদি) |  |  |  |  |
| (20) I felt scared without any good reason(a)-আমি কোনও যৌক্তিক কারণ ছাড়াই ভীত থাকতাম |  |  |  |  |
| (21) I felt that life was meaningless(d)- আমার মনে হয়েছিল জীবন অর্থহীন |  |  |  |  |

**The Pittsburgh Sleep Quality Index**

Instructions: The following questions relate to your usual sleep habits DURING THE PAST MONTH ONLY. Your answers should indicate the most accurate reply for the majority of days and nights in the past month. Please answer all questions. During the past month,

1. When have you usually gone to bed?-আপনি কখন ঘুমানোর জন্য বিছানায় যান? _________________________

2. How long (in minutes) has it taken you to fall asleep each night?-প্রতি রাতে শোবার পরে ঘুমাতে আপনার কতক্ষণ সময় (মিনিটে) লাগে?_______________________________

3. When have you usually gotten up in the morning?-আপনি সকালে কখন ঘুম থেকে উঠেন?________________

4. How many hours of actual sleep do you get at night? (This may be different than the number of hours you spend in bed)-রাতে আপনি প্রকৃতপক্ষে কত ঘন্টা ঘুমান? (আপনার বিছানায় কাটানোর সময়ের চেয়ে এটি ভিন্ন হতে পারে)

_______________________________

5. During the past month, how often have you had trouble sleeping because you.

|  | Not during the past month (0)-গতমাসে একবারও না (০) | Less than once a week (1)-সপ্তাহে একবারের চেয়েও কম (১) | Once or twice a week (2)- সপ্তাহে একবার বা দুইবার (২) | Three or more times a week (3)-সপ্তাহে তিনবার বা তারচেয়ে বেশি (৩) |
| --- | --- | --- | --- | --- |
| 5a. Cannot get to sleep within 30 minutes-৩০ মিনিটের মধ্যে ঘুমাতে পারি না |  |  |  |  |
| 5b. Wake up in the middle of the night or early morning-মাঝরাতে বা খুব ভোরে ঘুম থেকে জেগে উঠি |  |  |  |  |
| 5c. Have to get up to use the bathroom or washroom-বাথরুম বা ওয়াশরুম ব্যবহার করতে উঠতে হয় |  |  |  |  |
| 5d. Cannot breathe comfortably-স্বাভাবিকভাবে বা আরামে শ্বাস নিতে পারিনা |  |  |  |  |
| 5e. Cough or snore loudly-জোরে কাশি বা নাক ডাকি |  |  |  |  |
| 5f. Feel too cold-খুব বেশি ঠান্ডা লাগে |  |  |  |  |
| 5g. Feel too hot-খুব বেশি গরম লাগে |  |  |  |  |
| 5h. Have bad dreams-খারাপ স্বপ্ন দেখি |  |  |  |  |
| 5i. Have pain-ব্যথা হয় |  |  |  |  |
| 5j. Other reason(s) including how often you have had trouble sleeping because of this reason(s)-ঘুমে ব্যাঘাত ঘটার মত অন্যকোন কারন থাকলে তার স্কোর |  |  |  |  |

6. During the past month, how often have you taken medicine (prescribed or “over the counter”) to help you sleep?-গত মাসে আপনার ঘুমের জন্য আপনি কতবার ওষুধ (প্রেসস্ক্রিপশন অনুযায়ী বা কোন প্রেসস্ক্রিপশন ছাড়া) নিয়েছেন?

- Not during the past month (0)-গতমাসে একবারও না (০)
- Less than once a week (1)-সপ্তাহে একবারের চেয়েও কম (১)
- Once or twice a week (2)- সপ্তাহে একবার বা দুইবার (২)
- Three or more times a week (3)-সপ্তাহে তিনবার বা তারচেয়ে বেশি (৩)

7. During the past month, how often have you had trouble staying awake while driving, eating meals, or engaging in social activity?-গত মাসে কতবার গাড়ি চালানো, খাবার খাওয়া বা সামাজিক কার্যকলাপের সময় আপনার জেগে থাকতে সমস্যা হয়েছে?

- Not during the past month (0)-গতমাসে একবারও না (০)
- Less than once a week (1)-সপ্তাহে একবারের চেয়েও কম (১)
- Once or twice a week (2)- সপ্তাহে একবার বা দুইবার (২)
- Three or more times a week (3)-সপ্তাহে তিনবার বা তারচেয়ে বেশি (৩)

8. During the past month, how much of a problem has it been for you to keep up enthusiasm to get things done?-গত এক মাসে কোন কাজ করার উৎসাহ ধরে রাখতে আপনার কতটা সমস্যা হয়েছে ?

- Not during the past month (0)-গতমাসে একবারও না (০)
- Less than once a week (1)-সপ্তাহে একবারের চেয়েও কম (১)
- Once or twice a week (2)- সপ্তাহে একবার বা দুইবার (২)
- Three or more times a week (3)-সপ্তাহে তিনবার বা তারচেয়ে বেশি (৩)

9. During the past month, how would you rate your sleep quality overall?-গত এক মাসে আপনি আপনার ঘুমের ওভারঅল কোয়ালিটিকে কিভাবে রেটিং করবেন?

- Very good (0)
- Fairly good (1)
- Fairly bad (2)
- Very bad (3)
