# Supplementary material for: Sleep quality and psychological distress among Bangladeshi medical students: Prevalence, predictors, and sex-institutional differences
Source: Glob Epidemiol. 2026 Jan 6;11:100243. doi: 10.1016/j.gloepi.2026.100243 (PMC12818157; doi:10.1016/j.gloepi.2026.100243)
Supplement: Supplementary Material S2 [file mmc2.pdf]

Ref. JKKNIU.PS.Ethical.2023.60 Date: 09/12/2023

Abdul Muyeed
Assistant Professor
Department of Statistics
Jatiya Kabi Kazi Nazrul Islam University, Mymensingh, Bangladesh

Ethical Clearance

With reference to your application for ethical clearance, we are delighted to inform that your research proposal is entitled "**Prevalence and Associated Factors of Depression, Anxiety, Stress and Insomnia among Medical Students in Bangladesh: A Cross-sectional Survey**" has been received and approved by the research approval committee of Department of Population Science, Jatiya Kabi Kazi Nazrul Islam University. A panel of persons who review research proposals with respect to ethical implications and decides whether the additional actions need to be taken to assure the safety and rights of the study participants.

You are requested to follow the ethical guidelines mentioned below-

- Consent (Written or Oral) should be obtained from every respondent by explaining the objectives of the study and their benefits.
- The safety, rights and well being of the respondents should be protected.
- Identification of the respondents should not be disclosed to anyone who is not directly involved to the study.

This approval is provided based on the research proposal and it may be withdrawn in case of violation of any of the ethical guidelines prescribed by the committee.

09/12/2023

Md. Nure Alam Siddiqi
Assistant Professor & Head of the Department
Department of Population Science
Jatiya Kabi Kazi Nazrul Islam University, Bangladesh

CS CamScanner
